# Discussing the potential consequences of a diagnostic label before routine non-cancer screening: qualitative study with general practitioners and consumers

**DOI:** 10.1192/bjo.2025.5

**Published:** 2025-06-11

**Authors:** Rebecca Sims, Zoe A. Michaleff, Paul Glasziou, Rae Thomas

**Affiliations:** Institute for Evidence-Based Healthcare, Bond University, Gold Coast, Australia; Northern New South Wales Local Health District, Lismore, Australia; Tropical Australian Academic Health Centre, Ltd, Douglas, Australia

**Keywords:** Consequences, diagnostic labelling, general practitioners, health consumers, qualitative, screening

## Abstract

**Background:**

A diagnostic label can have harms and benefits, particularly when provided following routine health screening tests. Whether these are discussed in clinical encounters is unknown.

**Aims:**

To investigate whether potential impacts of diagnostic labelling are discussed before routine screening for non-cancer health conditions and explore the perceived value of such discussions by general practitioners (GPs) and healthcare consumers.

**Method:**

Eleven semi-structured interviews with GPs and two focus groups with eight consumers were conducted. Interviews and focus groups were audio-recorded, transcribed and analysed using thematic analysis methods based on framework analysis.

**Results:**

Prior to routine screening, most GPs did not discuss the potential consequences of diagnostic labelling, and no consumer recalled discussions of this nature. In contrast, many GPs provided information regarding the screening procedure and possible test limitations. Both GPs and consumers identified that it would be valuable to discuss the potential impacts of a diagnostic label; however, preferences varied as to the content and timing (i.e. before or after screening) of this discussion. Six themes that examine the utility of discussing the consequences of diagnostic labelling were identified: patient empowerment, patient variability, condition-specific information, GP and patient interactions and relationship, GP role and responsibilities, and characteristics of screening.

**Conclusions:**

The practice and perceived value of discussing diagnostic labelling consequences were recognised as important by both GPs and consumers. However, preferences regarding the content of discussions and whether these occurred in clinical encounters before or after screening varied.

## Non-cancer screening

Screening for health conditions is predicated on the principle that early detection of health anomalies provides access to earlier treatment, as well as increasing healthy behaviours and reducing risky ones, leading to positive health outcomes.^1,2^ However, this is not always the case. Screening occurs in individuals who are asymptomatic (without symptoms); however, when the screening outcome is above but close to the diagnostic threshold, subsequent diagnostic labelling (e.g. mild hypertension, mild hyperlipidaemia) potentially identifies otherwise healthy individuals as unwell.^2,3^ Although some researchers have proposed that screening may reduce clinical and economic burden, others have highlighted an increased burden owing to more individuals being labelled and the potential for those labelled to experience negative psychological, psychosocial and physical consequences following screening and subsequent diagnostic labelling.^1–6^ Evidence for the impacts of cancer screening (e.g. breast cancer, prostate cancer) and subsequent diagnostic labelling and treatment has received significant research attention.^4,5^ However, routine non-cancer screening – that is, screening for physical (e.g. hypertension, hyperlipidaemia) and psychological (e.g. depression, anxiety) non-cancer health conditions – has received less research focus.

## Diagnostic labelling

The use of diagnostic labels has been found to be increasing.^6,7^ This trend is likely to be influenced by screening and changes in diagnostic criteria, including the expansion of disease definitions to encompass mild or lower thresholds for health conditions.^6,8,9^ The impacts of diagnostic labelling on individuals and healthcare services range from positive (e.g. relief, self-understanding) to negative (e.g. psychological distress, anxiety, negative side-effects of treatment) and include financial impacts due to diagnostic cascades and overtreatment.^8,10^ A recent systematic review found that anxiety increased in the short term following health condition screening; however, longer-term consequences were unclear.^11^ Further, social constructionism emphasises the role of society and social interactions in developing and maintaining routine screening and diagnostic labelling, as well as stereotypes and perceptions of capabilities of individuals with a diagnostic label.^2,12–14^ Despite the potential impacts of screening and subsequent condition-labelling, even in their mildest form, it is not known whether or how general practitioners (GPs) discuss these complex issues with patients or whether patients are aware or adequately informed of the potential consequences of screening.

## Objectives

From the perspective of GPs and healthcare consumers (consumers), we aimed to identify whether GPs and consumers discussed the potential consequences of diagnostic labelling before screening and the applicability of the current literature in the clinical encounter. The research questions for this study were as follows.

- Do GPs discuss the potential consequences of diagnostic labelling before routine screening for non-cancer health conditions? If so, why and how, and if not, why not?
- What is the applicability of the current literature to the consequences of diagnostic labelling before non-cancer screening?

## Methods

The study protocol is available on Open Science Framework (https://osf.io/3fxvn/). The authors assert that all procedures contributing to this work comply with the ethical standards of the relevant national and institutional committees on human experimentation and with the Helsinki Declaration of 1975, as revised in 2013. All procedures involving human subjects and/or patients were approved by Bond University Human Research Ethics Committee (RS00318 and RS00322).

### Participants and recruitment

The sample size required for qualitative studies using thematic analysis has been suggested to be 6–10 interviews and 2–4 focus groups.^15^ However, the stopping criteria are based on data saturation or the non-emergence of new themes, rather than achieving a specific number of interviews or focus groups.^15^

#### General practitioners

Eligible GPs were those currently practising as GPs in Australia. Recruitment strategies included advertising through mailing lists, websites and social media accounts of professional organisations (e.g. GoldNet Research) and snowballing. Interested participants completed an online survey, which included written consent and eligibility and demographic questions. Eligible participants were contacted by R.S. to schedule the semi-structured interview. GPs received a AU$100 gift voucher as reimbursement for their time.

#### Healthcare consumers

Consumers were purposely sampled to align with the Royal Australian College of General Practitioners recommendations for preventive age-related health checks for individuals aged 45–65 years.^16^ We recruited consumers aged 40–65 years who were currently or soon would be eligible for these checks.^16^ As we were interested in discussing the consequences of diagnostic labelling following screening, we included consumers who had not been diagnosed with cancer or health conditions requiring intensive treatment. We excluded consumers receiving treatment for a long-standing or life-threatening health condition (e.g. chronic kidney disease, cardiovascular disease), undergoing testing for a suspected health condition, unable to provide informed consent, unable to speak or understand English, or unable to access a computer and reliable internet connection.

Recruitment strategies included advertising through mailing lists, websites and social media accounts of consumer organisations (e.g. JoinUs) and snowballing. Interested participants completed an online survey, which included eligibility checking, written consent and demographic questions. Eligible participants were contacted by R.S. to be allocated to a focus group. Consumers received a AU$50 gift voucher as reimbursement for their time.

### Procedure and materials

Data were collected through semi-structured interviews with GPs and focus groups with consumers. Different data collection methods were used owing to challenges in coordinating multiple GPs to attend a scheduled focus group.^15^ Semi-structured interviews and focus group structures, interview guides and presentation materials were developed in consultation with the wider research team; additional information is provided in Supplementary Table 1 available at https://doi.org/10.1192/bjo.2025.5.

#### Semi-structured interviews with GPs

R.S. conducted semi-structured interviews of up to 1 h duration between May and July 2023 via video-enabled online platforms (i.e. Zoom and Microsoft Teams). GPs were asked open-ended questions regarding their clinical practice, presented with a short pre-recorded presentation on available research evidence about the consequences of diagnostic labelling (recorded by R.S. and available at https://osf.io/yp5wz), offered the opportunity to comment on the presentation and asked to discuss the clinical applicability of the information presented.

#### Focus groups with consumers

Focus groups, each 90 min in duration, were conducted in August 2023 via Zoom and were facilitated by R.S. and R.T. Consumers were presented with two short, pre-recorded presentations. The first provided overviews of routine screening and interpretation of risks for health conditions (recorded by P.G. and available at https://osf.io/75mpa). The second was the same as that presented to GPs. After each presentation, consumers were offered the opportunity to discuss the information presented and ask questions. Consumers were then asked open-ended questions to facilitate discussion regarding the applicability of the information to screening.

### The research team

Adhering to the Consolidated Criteria for Reporting Qualitative Research (COREQ) checklist, the study team have expertise across psychology, clinical medicine, clinical epidemiology and public health. R.S. is a clinical psychologist and PhD candidate, with an interest in the impacts of diagnostic labelling. Z.A.M. is a physiotherapist with a PhD and an interest in evidence-based assessment, diagnosis and treatment of health conditions. P.G. is an academic general practitioner and clinical epidemiologist with a PhD and leads international research on overdiagnosis and overtreatment. R.T. is a psychologist with a PhD and an interest in consumer and community involvement in healthcare and policy development. R.S., Z.A.M. and R.T. are female, and P.G. is male. The completed COREQ checklist is provided in Supplementary Table 2.

### Analyses

Demographic data were collated, summarised and presented using descriptive statistics. Data from semi-structured interviews with GPs and focus groups with consumers were audio recorded and transcribed verbatim using automated transcription. Transcripts were checked for accuracy by R.S. We used thematic analysis methods based on framework analysis, as described by Ritchie and colleagues.^15^ Social constructionism underpins the theoretical framework, whereby the meanings produced through research are influenced by the social world of both the participants and researchers.^12,13,15^ Subsequently, we aimed to understand the diversity of participants’ experiences, rather than identifying one uniform meaning.

Transcripts were analysed using NVIVO version 12 (Lumivero; see https://lumivero.com/products/nvivo/). An inductive and iterative thematic approach was used to facilitate understanding of responses and participant perspectives. First, data familiarisation involved transcript review and development of an initial coding framework (R.S.), analysing responses to each question and collective responses across transcripts. Through discussion and feedback from the wider research team, the initial framework was refocused on how the data addressed the specific research questions, and the number of themes was reduced to reflect the data more accurately. R.S. then re-coded two transcripts to the revised framework. This coding was discussed among the wider research team, with the overall themes and subthemes remaining unchanged following discussion. The framework was then applied to the whole data-set (R.S.), and data saturation was achieved. Last, the final coding was reviewed by R.T. and/or Z.A.M. to ensure reliability.

### Transparency declaration

The authors confirm that this manuscript is an honest, accurate and transparent account of the study reported; that no important aspects of the study have been omitted; and that any discrepancies from the study protocol have been explained.

### Patient and public involvement

No patients or members of the public were involved in the conceptualisation of this study. GPs and consumers took part in the conduct of this study as participants but were not involved in the study design or the analysis or write-up of results.

## Results

### Demographics

#### General practitioners

Thirteen GPs expressed interest in participating, and 11 participated. The 11 GPs had an average of 13 years practising as a GP (range 4–27 years), and six of them were female (55%). Most GPs (*n* = 10, 91%) practised in metropolitan locations, and roughly half (*n* = 6, 55%) worked in GP-only practices and across multiple patient demographics. One eligible GP failed respond to initial researcher contact following expression of interest, and another failed to attend their scheduled semi-structured interview and did not respond to subsequent contact. Table 1 provides additional information regarding GPs.

**Table 1:** General practitioner (GP) demographics

Demographics	GPs (*N* = 11)
Female, *n* (%)	6 (55)
Years practising as GP, mean (range)	12.6 (4–27)
Additional specialisation^a^, *n* (%)	3 (27)
Location^b^, *n* (%)	
Metropolitan	10 (91)
Rural	1 (9)
Clinical setting, *n* (%)	
GP-only practice	6 (55)
Multidisciplinary practice	3 (27)
Hospital and GP-only or multidisciplinary practice	2 (18)
Predominant patient demographic^c^, *n* (%)	
Infants and children (0–12 years)	4 (36)
Adolescents (13–18 years)	3 (27)
Young adults	6 (55)
Women’s health	7 (63)
Men’s health	4 (36)
Older adults	6 (55)

a. Areas of additional specialisation were psychiatry and research, Diploma of Child Health, rural generalism, and Fellow of the Royal Australian College of General Practitioners.

b. Location based on the modified Monash model.^17^

c. GP could practise across more than one patient demographic.

#### Healthcare consumers

Eleven consumers expressed interest in participating, and eight participated. Six of the eight (75%) consumers were female and married or in a de facto relationship, and the average age was 55 years (range 46–63 years). Four (50%) lived in a regional location and were highly educated (university postgraduate degree). Four (50%) reported having undergone screening for a health condition, and most (*n* = 7, 87.5%) reported having a diagnosed health condition (e.g. asthma, coeliac disease, hypertension) detected more than 2 years ago. In addition, one consumer did not respond to initial researcher contact after an expression of interest, and two failed to attend their scheduled focus group and did not respond to subsequent contact. Table 2 provides additional information regarding consumers.

**Table 2:** Healthcare consumer demographics

Demographics	Focus group 1 (*n* = 4)	Focus group 2 (*n* = 4)	Total (*N* = 8)
Female, *n* (%)	4 (100)	2 (50)	6 (75)
Age in years, mean (range)	52 (46–63)	57.8 (51–62)	54.9 (46–63)
Cultural background, *n* (%)			
Australian	2 (50)	1 (25)	3 (37.5)
Australian and British	1 (25)	1 (25)	2 (25)
Australian and Dutch	1 (25)	–	1 (12.5)
Australian and Italian	–	1 (25)	1 (12.5)
South American and Italian	–	1 (25)	1 (12.5)
Location^a^, *n* (%)			
Metropolitan	–	3 (75)	3 (37.5)
Regional	4 (100)	–	4 (50)
Rural	–	1 (25)	1 (12.5)
Marital status			
Single	–	1 (25)	1 (12.5)
Married or de facto	4 (100)	2 (50)	6 (75)
Divorced	–	1 (1)	1 (12.5)
Education			
Finished high school or equivalent	–	1 (25)	1 (12.5)
Some university or TAFE	–	2 (50)	2 (25)
Undergraduate or TAFE graduate	1 (25)	–	1 (12.5)
Postgraduate degree	3 (75)	1 (25)	4 (50)
Ever undergone screening for a non-cancer health condition^b^, *n* (%)	3 (75)	1 (25)	4 (40)
Previously received a non-cancer diagnosis^c^, *n* (%)	3 (75)	4 (100)	7 (87.5)

TAFE, Technical and Further Education.

a. Location based on the modified Monash model.^17^

b. Reported non-cancer screening included blood pressure and diabetes.

c. Reported non-cancer diagnoses included asthma, coeliac disease, rheumatoid juvenile arthritis, gestational diabetes mellitus, hypertension, vasculitis, thalassemia trait, gastroesophageal reflux disease and narrow-angle glaucoma (with all reported diagnoses made more than 2 years ago).

### Qualitative synthesis

When asked whether GPs discussed the potential impacts of diagnostic labelling before screening (research question 1), there was little support for discussing specific impacts of diagnostic labelling before a condition was identified. However, we identified themes regarding general information GPs included in conversations before screening. Whether the literature on the consequences of diagnostic labelling was applicable to their GP–patient encounters (research question 2), qualitative themes related to the value of discussions being routine or only when a health condition was identified. Overall, the two research questions were addressed through six themes: patient empowerment, patient variability, condition-specific information, GP and patient interactions and relationship (four subthemes), GP role and responsibilities (four subthemes), and characteristics of the screening test (two subthemes).

**Fig. 1:**
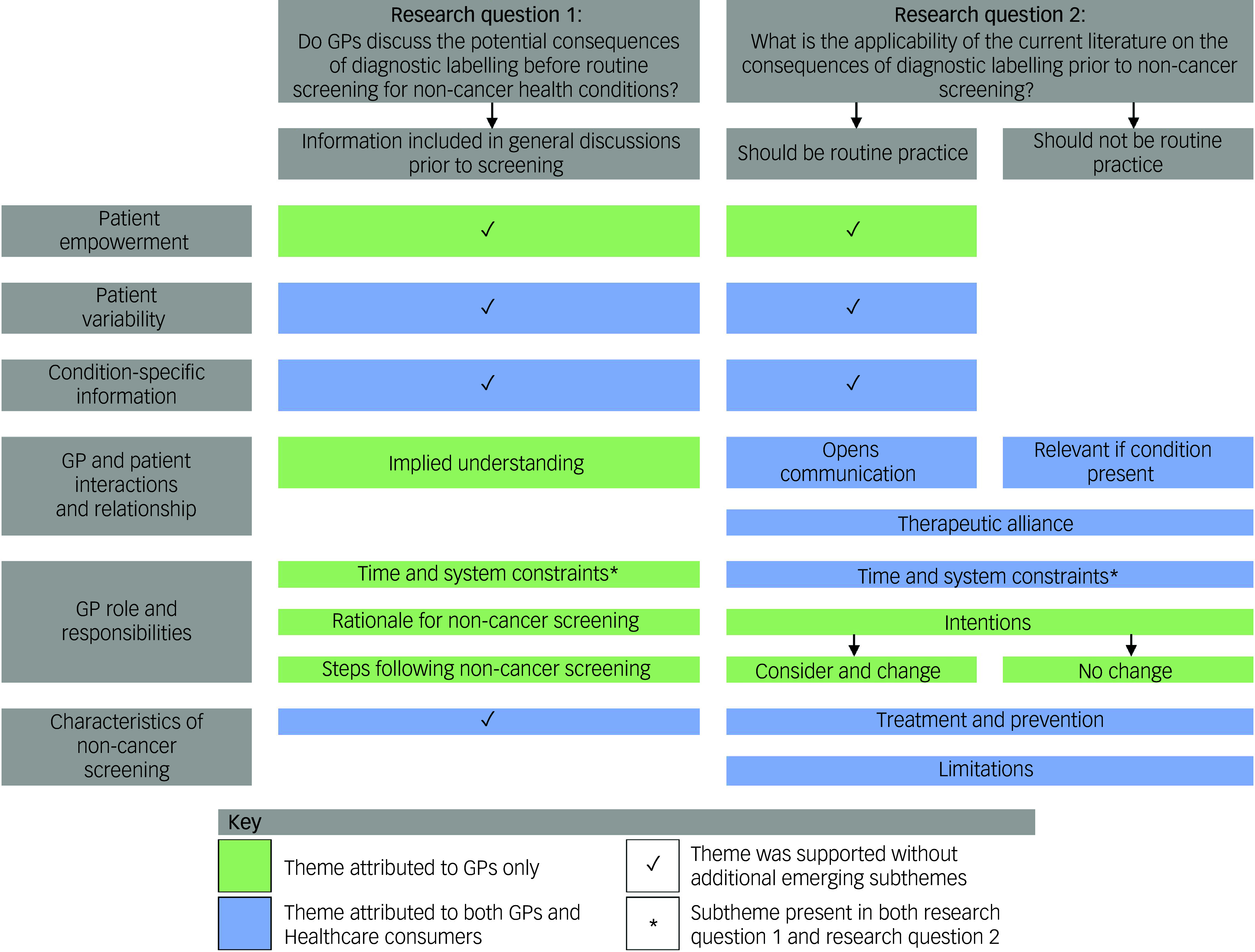
Relationships among themes, subthemes and research questions supported by general practitioners and/or consumers. GP, general practitioner.

Table 3 defines themes and subthemes, and Fig. 1 provides a representation of the relationship between themes and research questions. Quotes from participants are attributed to group and characteristics. GPs are acknowledged by their participant number, sex (F, female; M, male), years of clinical experience and location (e.g. GP1, M, 4 years, metropolitan). Consumers are designated by their participant number, sex (F, female; M, male), previously diagnosed health condition (yes, no) and location (e.g., C1, F, yes, regional).

**Table 3:** Theme and subtheme descriptions

Patient empowerment	
Discussions related to the consequences of diagnostic labelling are an opportunity to improve patients’ understanding of the health condition, provide guidance on lifestyle modifications, and empower patients to have control of their health and healthcare	
Patient variability	
Information is tailored to the patient, providing information relevant to the individual, their context, history, level of understanding and desired level of information.	
Condition-specific information	
Information needs to be specific to the health condition and screening being conducted.	
General practitioner (GP) and patient interactions and relationship	
Importance of GP and patient communication and relationship to increase engagement, challenge preconceived ideas, provide education and information relevant to the patient, and extend patient understanding.	
Implied understanding	GP belief that patients understand non-cancer screening as routine
Open communication	Discussion of information should be completed before non-cancer screening, as this invites open dialogue between patient and GP, including provision of important health information and education
Relevant if condition present	Discussion of information is not valuable before non-cancer screening as it exceeds what might be required and what can be understood by patients, particularly for mild health conditions; however, discussions would become relevant if a condition is identified through non-cancer screening
Therapeutic alliance	Contribution of the therapeutic alliance to discussions regarding non-cancer screening and subsequent provision of results
GP role and responsibilities	
Perceived role of the GP, including requirements and understanding of GP practice, system requirements, changes over time and/or with experience, and assumptions.	
Rationale for non-cancer screening	Provide an explanation for what the non-cancer screening process entails and why it is important
Steps following non-cancer screening	Potential next steps, if a condition is identified through non-cancer screening, are discussed
Time and system constraints	Time limitations and system and/or workplace requirements inform and impede practices, including when and how discussions might be achieved
Intentions: consider and change	Consideration of and potential changes to communication practices before non-cancer screening
Intentions: no change	Currently engage in considered practice and do not believe changes are required
Characteristics of non-cancer screening	
Non-cancer screening provides an opportunity to identify and treat health difficulties, with the goal of preventing more serious health difficulties; however, test reliability and requirements may also pose risks and limitations.	
Treatment and prevention	Perceived opportunities and benefits regarding non-cancer screening, including the opportunity to identify and treat elements of health, with the goal of prevention of more serious health difficulties
Limitations	Perceived limitations and challenges to non-cancer screening and discussion before screening, including test limitations and patient motivation for presentation to GP

### Research question 1: Do GPs discuss the potential consequences of diagnostic labelling prior to routine screening for non-cancer health conditions? If so, why and how, and if not, why not?

Most GPs said they did not discuss potential impacts of diagnostic labelling before routine screening. However, many reported that they had brief conversations, centred on the screening procedure and possible limitations, with patients before screening. Other than screening during pregnancy, consumers struggled to identify health conditions for which they might be screened and could not recall GPs discussing potential impacts of diagnostic labelling before screening. All six themes were identified in relation to this research question (Fig. 1), with subthemes within the GP role and responsibilities (time and system constraints, rationale for non-cancer screening, steps following non-cancer screening) and GP and patient interactions and relationship (implied understanding) themes. Three themes were reported by GPs only (patient empowerment, GP and patient interactions and relationship, and GP role and responsibilities), and three were reported by both GPs and consumers (patient variability, condition-specific information, and characteristics of non-cancer screening). Themes are discussed below and detailed in Table 4.

**Table 4:** Do general practitioners (GPs) discuss the potential consequences of diagnostic labelling before routine screening for non-cancer health conditions? If so, why and how, and if not, why not?

Themes and subthemes	Illustrative comments
Patient empowerment	
	‘I think it’s important that the patient is educated or has a bit of autonomy and the ability to look into that further. But I think it’s important that they also know that it is considered a low risk or a benign condition… I think if you present it correctly, you can mitigate those a lot of the time if you just say, “look, do you have any other questions?” We’re just trying to make it as easy for them to digest.’ (GP7, M, 5 years, metropolitan) ‘Educating them, is important, without alarming them… When we’re talking about a mild non-cancer diagnosis, I would like to think that they feel empowered about their own health. That they can be in charge of implementing some preventative strategies and have a little bit more control over the next few years.’ (GP9, F, 27 years, metropolitan)
Patient variability	
	‘You sometimes need to target the screening to the patient. So, if you’ve got a very, very anxious patient, then you may err on the side of not screening if you think the harm of their anxiety is going to be greater than the benefit of the screening… I target it at the patient. There are some people who I know will be fine, based on just my knowledge of them.’ (GP3, F, 20 years, metropolitan) ‘If I go to the doctor now, I’m either going because there is some sort of discomfort that I’ve got and then some sort of test is being done.’ (C3, F, yes, regional)
Condition-specific information	
	‘I guess that really depends on exactly what you’re screening for, because I think the harms and the benefits are very condition specific.’ (GP5, F, 10 years, metropolitan) ‘Not besides when I was pregnant, they’re [the doctors] like, “Whoa, if you go ahead with this, then this is what it could be”. There was a lot more of it in my pregnancy probably.’ (C2, F, no, regional)
GP and patient interactions and relationship	
Implied understanding	‘It’s something I’d say probably isn’t done particularly commonly. Most people are of the understanding that it’s just a routine blood test and I’d probably say a lot of that counselling actually happens after the abnormal finding is raised… I think that’s kind of an implied understanding that it’s a routine thing.’ (GP7, M, 5 years, metropolitan) ‘There’s an inherent consent. You get the blood pressure cuff, you start moving towards them with it, and they put their arm out, and they’ve made an appointment, and they’ve waited for the appointment to roll around, and there they are. They vote with their feet. I think there’s an inherent consent in the fact that they’re attending the appointment.’ (GP9, F, 27 years, metropolitan)
GP role and responsibilities	
Time and system constraints	‘I also explain that the radiologists have to say everything that’s on the scan and they sometimes say this would benefit from follow up, but it’s mainly just to cover themselves for the very, very small percentage of cases that do need following up.’ (GP3, F, 20 years, metropolitan) ‘You’re time poor… So, a lot of the time it’s just, “Here’s the blood form. We need to get this done”.’ (GP7, M, 5 years, metropolitan)
Rationale for non-cancer screening	‘It’s a privilege to be able to do screening test in terms of we have a health care system that can take on that…and trying to detect conditions early so that way we can prevent complications and long-term consequences and therefore the ultimate goal living longer, healthier, happier lives… “We’re looking after your health and well-being and we’re trying to make sure that we detect things before they become a problem and you can try to intervene if possible before they do”.’ (GP2, F, 5 years, metropolitan) ‘I guess you just explain what you’re doing and why you’re doing it. So, I try and normalise the process during consults… telling patients why doing it ultimately and judging their resistance if there is a bit of resistance, then rolling with it. But I mean, more often than not, them understanding why you’re doing it is enough.’ (GP6, M, 6 years, metropolitan)
Steps following non-cancer screening	‘We might get a false positive in which case we need to repeat a test, or we might need to do further investigation. We might have to see a specialist. And yes, we’re doing it to prevent illness, but it also might mean that there’s a cost to you and we might find things that we didn’t need to know about.’ (GP4, F, 5 years, metropolitan) ‘And talking about the benefits, would be talking about possible treatments that might be available if conditions are recognised.’ (GP5, F, 10 years, metropolitan)
Characteristics of non-cancer screening	
	‘I do a little spiel in terms of explaining, the best I can to patients, that there’s always limitations with screening tools. They’re not a hundred percent perfect. We will miss things and we will overdiagnose things.’ (GP2, F, 5 years, metropolitan) ‘Non-cancer screening can be as simple as screening someone’s mental health. And it’s harder to describe the potential downsides of that before you ask someone how they’re feeling.’ (GP11, M, 22 years, rural) ‘[During pregnancy] was the only time where I think someone talked about the importance of this test or whether I did it or didn’t do it and what the benefits or disadvantage of that prior.’ (C3, F, yes, regional)

#### Patient empowerment

GPs discussed the importance of empowering patients though provision of information to aid patients’ ability to make an informed decision before undergoing screening. GPs acknowledged the importance of providing information and an opportunity for the patient to ask questions and consider lifestyle changes.

#### Patient variability

Both GPs and consumers discussed the need to target screening discussions and information provision to the requirements and preferences of the individual patient. GPs reported that they tailored the information provided to patients based on a range of factors including their clinical acumen.

#### Condition-specific information

Similarly, GPs and consumers discussed the information provided being specific to the condition being screened.

#### GP and patient interactions and relationship

GPs discussed the importance of GP and patient relationships in facilitating patient engagement and understanding. However, some GPs stated that patients understood screening as routine (implied understanding); therefore, they did not ordinarily have discussions about labelling before screening.

#### GP role and responsibilities

Only GPs raised this theme in relation to discussing the potential impacts of diagnostic labelling. Three subthemes were identified. The time and system constraints subtheme highlighted that clinical encounters are often guided by time limitations and workplace regulations. The rationale for non-cancer screening subtheme centred around general discussions about screening tests rather than the impact of diagnostic labelling, whereas the steps following screening subtheme centred on managing patient impact after test results were provided.

#### Characteristics of non-cancer screening

GPs highlighted limitations and challenges associated with screening tests. Many comments about screening involved minimally invasive tests which provided an opportunity to identify, treat and prevent more serious health difficulties. However, challenges associated with test reliability were also raised. Consumers did not recall any discussions about the potential impact of having a diagnostic label except during pregnancy.

### Research Question 2: What is the applicability of the current literature on the consequences of diagnostic labelling before non-cancer screening?

Following the presentation of information, most GPs and consumers said that conversations regarding the consequences of diagnostic labelling ‘should be routine practice,’ with this view represented across all six themes. However, others perceived these discussions as relevant only if a condition was identified through screening (‘should not be routine practice’). This latter viewpoint was supported in three themes (GP and patient interactions and relationship, GP role and responsibilities, and characteristics of non-cancer screening). For all themes except patient empowerment, both GP and consumer viewpoints were reported (Fig. 1). Within the GP roles and responsibilities theme the subtheme of time and system constraints was again identified, with GPs also mentioning their intentions regarding changes to clinical practice (GP intentions). Subthemes also emerged for GP and patient interactions and relationship (opens communication, relevant if condition present, therapeutic alliance) and characteristics of non-cancer screening (treatment and prevention, limitations). Themes are discussed below and detailed in Table 5.

**Table 5:** What is the applicability of the current literature on the consequences of diagnostic labelling prior to non-cancer screening?

Themes and subthemes	Illustrative comments
Patient empowerment	
	‘It reinforces more general health views that I have, that people will have all manner of reactions to diagnosis and how well that is managed in the first instance will actually set up a trajectory that matters. So doing it really well from the beginning may actually change the trajectory of someone’s experience of whatever that condition might be.’ (GP1, M, 4 years, metropolitan) ‘I mean, obviously it would be the more information the better. We want the patients to be empowered and have more understanding. I think it would probably help them contextualise their responses a little bit more as well.’ (GP7, M, 5 years, metropolitan)
Patient variability	
	‘People vary the amount of information they want… I’m not going to force information on somebody who doesn’t wish it. Equally people who wish information very much deserve to have it.’ (GP8, M, 25 years, metropolitan) ‘It’s really hard to put the general population in the box, right? I guess it just depends on the person. There’s one person who’s going to want to dig deep into it and another person who’s just too busy with their life and just want to know what they need to know and move on. So, it’s hard.’ (C7, F, yes, metropolitan)
Condition-specific information	
	‘[Regarding the relevance of discussions prior to screening] Broadly? Probably not. Some diabetes screening, cardiovascular screening, MSK [musculoskeletal] screening maybe. Some MSK stuff possibly, but probably not. But then neurodevelopmental in kids, for example, or infection screenings, then potentially yes.’ (GP6, M, 6 years, metropolitan) ‘Being labelled as asthmatic versus epileptic might be different. And I would suspect that diabetes versus ADHD [attention-deficit hyperactivity disorder] are different labels and perceived very differently.’ (C4, F, yes, regional)
GP and patient interactions and relationship	
Opens communication	‘It has to be routine because we are potentially altering how someone makes meaning of their life, and I really don’t know what’s more powerful in healthcare. There’s no bigger responsibility in healthcare than respecting and being humbled by the fact that what we do will change the way someone experiences living. And so, we need to be very aware, but we also need to invite dialogue about that with patients. Because perhaps they don’t quite understand how profound these things can be, and we’re the professionals, we’re supposed to know.’ (GP1, M, 4 years, metropolitan) ‘Do I want to be told everything? I’m a very curious person, so maybe I’m an outlier. I want to know everything. So, you’ve got to tell me as much as you can tell me so that I can decide.’ (C2, F, no, regional)
Relevant only if condition present	‘It might actually compound anxiety if there’s just too much to take on. Let’s wait and see if we’ve got it or not before talking about the consequences of diagnosis.’ (GP9, F, 27 years, metropolitan) ‘I think for me the point of information, is that I want it at the labelling point… I think if I was to come back with those tests results and at that point where I’m maybe being given the label or being told this is possibly now what we’re looking at, this is the next level of testing, that’s the point I would want lots of information to make my decision.’ (C3, F, yes, regional)
Therapeutic alliance	‘I guess you just get a feel of it over the years of knowing them. Others which are not regular patients, no, you’d have no idea. And that’s where it’d be a little bit harder. But certainly, the ones that we get to know quite well. You’ve got quite a good idea of how things like that will impact on them.’ (GP5, F, 10 years, metropolitan) ‘I also had a very, very communicative practitioner who was able to explain to me, in terms that I easily understood, how it all works and what can happen. And he’s very very very good at what he does. And that was able to make me feel very safe.’ (C8, F, yes, metropolitan)
GP role and responsibilities	
Time and system constraints	‘For me the barrier is time. If we’re going to fit that in, realistically, what else are we going to push out of the consult? […] We’re going to have to try and move something else out. Or the patient’s going to have to come back for another consult and for the patients, that’s funding the time themselves, but also, we’re not a bulk build practice. So, it’s also then the cost associated with that for them as well.’ (GP5, F, 10 years, metropolitan) ‘I think that due to time restraints it’s often abandoned, and we’ll go down that road when we get the results, maybe. “Let’s just see what’s happening” they usually say or “This is routine, do you mind having some tests”.’ (C1, F, yes, regional)
GP intentions: consider and change communication practices	‘It’s probably reinforced something I’ve always thought was important, but maybe in the interests of time, I sometimes might curtail. So, it just reminds me that it is quite important to make sure these conversations are had, even if I think I do them routinely, there’s probably room for improvement.’ (GP1, M, 4 years, metropolitan) ‘Specifically what tests I do when, or what screening I do when, probably not changing. But being conscious of how I communicate things to patients, probably yes.’ (GP6, M, 6 years, metropolitan)
GP intentions: no change	‘I’m not sure if I would do things differently, because I feel I already take that into consideration.’ (GP10, F, 10 years, metropolitan) ‘Honestly, in the interest of time, I do not think I would make any changes before I did it. After, I might try and not use a label, but try and use something lifestyle based, and for what we’re going to do for preventative health in the future rather than trying to give them a label as such. A label is useful, I think, if it is going to give them access to services and treatment.’ (GP4, F, 5 years, metropolitan)
Characteristics of non-cancer screening	
Treatment and prevention	‘To my very core, I believe in preventive medicine. So, I think the sooner that something can be diagnosed, if it’s there, the sooner we can get on with early measures of treatment and then the better the outcome, usually.’ (GP9, F, 27 years, metropolitan) ‘Personally, I think I would want to know. Because I think I would do something about it.’ (C6, M, yes, rural)
Limitations	‘I think perhaps sometimes it brings up almost false positive findings, and that a lot of people if you ask them “do you feel tired?”, the answers are almost always going to be yes. So, you might be then investigating when it’s not possibly necessary. But other than that, I don’t think there’s any harms.’ (GP5, F, 10 years, metropolitan) ‘If you start screening everybody who just feels normal, they’re bound to find something, eventually. I mean, our body functions in weird ways so the more you dig the more you find.’ (C5, M, yes, metropolitan)

#### Patient empowerment

Some GPs noted that discussions before screening would be valuable to facilitate patient understanding of and receptiveness to information in the future, even if the information discussed was not immediately relevant.

#### Patient variability

Regarding the potential of a diagnostic label to be useful in discussions before screening, both GPs and consumers considered differences in individual patient preferences for information and the need to adapt to these preferences.

#### Condition-specific information

GPs and consumers noted that discussions before screening needed to contain information specific to the condition being screened, with differences between physical and psychological health conditions also identified.

#### GP and patient interactions and relationships

Some GPs and consumers perceived discussions regarding the consequences of diagnostic labelling before screening as valuable and considered that they should be routine practice. Reasons for this included the potential to invite dialogue between the GP and patient and provide an opportunity to convey important health information and education (opens communication). Other GPs and consumers perceived discussions regarding the consequences of diagnostic labelling as not valuable and considered that they should not be routine practice before screening, as such concepts might be difficult to understand and exceed requirements. However, these GPs and consumers added that discussions would be necessary after screening if conditions were identified.

Regardless of whether discussions were preferred as routine practice or only if conditions were identified, the contribution of the therapeutic alliance to the clinical encounter was emphasised by GPs and consumers, with a stronger therapeutic alliance suggested to increase GP ease of, and patient response to, communication.

#### GP role and responsibilities

Time and system constraints were again identified as challenging with respect to how and when routine discussions about the consequences of diagnostic labelling might occur, with these difficulties raised irrespective of whether GPs and consumers considered that discussions should be routine or not.

Regarding intentions, from the information provided and discussed in the semi-structured interviews, many GPs noted that they would consider and change conversations prior to screening. Specifically, GPs stated that they would be more conscious of potential impacts of screening, including diagnostic labelling, and allow increased time to have discussions with patients before screening. Other GPs noted that *no change* would be made to screening practices or discussions before this, mostly because they perceived their current practices to include sufficient discussions or because of time constraints.

#### Characteristics of non-cancer screening

Two subthemes emerged in relation to the value of discussing potential impacts of diagnostic labelling, with these identified as important in both routine and non-routine conversations. Both GPs and consumers discussed screening as an opportunity to identify and treat elements of health and prevent more serious health complications (treatment and prevention). Further, possible limitations to screening were identified, including test limitations, over-investigation, financial requirements and the potential to overwhelm patients (limitations).

## Discussion

We conducted 11 semi-structured interviews with GPs and two focus groups with eight consumers to examine whether the potential consequences of diagnostic labelling were discussed before routine screening and to identify the perceived value of such evidence-informed discussions. Many GPs reported that they provided patients with brief information regarding screening procedures and limitations before routine screening; however, no GPs reported discussing potential consequences of diagnostic labelling. Similarly, consumers could not recall GPs discussing potential consequences of diagnostic labelling before screening, except during pregnancy. The perceived value of discussing consequences of diagnostic labelling before screening varied. Some GPs and consumers considered that these types of discussion would facilitate understanding, whereas others thought they would only be valuable after a condition had been identified. Some GPs noted that they would consider making changes to their clinical practice to incorporate these labelling discussions before screening, whereas others stated that no changes were required. Six overarching themes related to the value of discussing the consequences of diagnostic labelling were identified: patient empowerment, patient variability, condition-specific information, GP and patient interactions and relationship, GP role and responsibilities, and characteristics of non-cancer screening.

### Strengths and limitations

This study provided insights from the two perspectives present in the clinical encounter: GP and consumer. This allowed comparison of perceptions between two populations who either impact (GPs) or are impacted by (consumers) screening and highlighted differences and similarities in perceptions of the information discussed. Conducting both semi-structured interviews and focus groups was also a strength, as it was intended to facilitate greater engagement from both populations. Multiple recruitment techniques were used to broaden the potential participant pool and increase the diversity of perspectives. Provision of pre-recorded presentations ensured consistency of information presented, and the use of standardised interview guides enabled targeted, but flexible, discussions.

Several limitations potentially affected our results. Difficulty recruiting both GPs and consumers resulted in participant numbers substantially lower than those anticipated for both semi-structured interviews and focus groups. Although thematic saturation was achieved across modalities and populations, additional findings may have emerged if further focus groups with greater participant numbers had been completed. Homogeneity of recruited participants, including geographical similarities, may have affected the diversity of themes; it is thus important to consider the applicability of the developed themes to rural, remote or underprivileged populations and to consumers with low literacy. The online format of both semi-structured interviews and focus groups, while potentially increasing accessibility, may have deterred some individuals from participating and affected the level of engagement of those who participated.

### Results in relation to existing studies

Our results highlight variability in patient preferences regarding discussions of diagnostic labelling and a need for GPs to be aware of or quickly ascertain a patient’s informational needs and preferences. Similar patient variability has been found in research examining medical maximising and minimising in healthcare preferences, whereby medical maximisers prefer active healthcare (e.g. optional medical tests and treatments), whereas medical minimisers prefer passive healthcare (e.g. medical tests and treatments only when necessary).^18^ In addition to patient factors, our findings emphasise the importance of GP–patient interactions and relationship (including therapeutic alliance) in facilitating when and how discussions are completed, as well as patients’ feeling understood and respected in the communication. Similarly, previous research supports therapeutic alliance and GP–patient relationships as important to patient satisfaction, treatment adherence and clinical outcomes.^19–23^

In the current sample, discussions regarding the potential consequences of a diagnostic label before screening were not ordinarily completed, and both GPs and consumers appreciated patient preferences and health conditions as important considerations. However, our findings parallel results of research examining the communication of test results in primary care, with one study finding that patient characteristics (e.g. anxiety, health literacy) and health condition characteristics (e.g. severity) influenced how and when results were communicated.^24^ Previous research has suggested a tendency for both GPs and patients to overestimate the benefits and underestimate the harms of screening.^25,26^ The potential to underestimate the harms of screening was further highlighted by the findings of this study, which suggest that some GPs may overlook potential harms of screening in favour of potential benefits. One such overlooked harm may be the possibility that unnecessary testing, resulting from screening, might itself be harmful. We also found that the language used by GPs, particularly when discussing the minimal invasiveness of screening tests, echoes the underestimation of potential consequences of being given a diagnostic label.

A systematic review of both qualitative and quantitative studies examined the barriers and facilitators to prevention (e.g. through screening and/or addressing lifestyle change) of cardiometabolic diseases (e.g. diabetes mellitus, chronic kidney disease) in primary care.^27^ The review found GP time restrictions and workload to be among the most frequently reported barriers, whereas strong GP–patient relationships and appreciation of the importance of prevention were frequently reported facilitators of screening and prevention.^27^ Although it did not focus on the consequences of diagnostic labelling, the review echoed our findings, highlighting time and workload as barriers to addressing asymptomatic health conditions in primary care. Additional discussions regarding the consequences of diagnostic labelling may inflate this barrier, with further consideration of how and when to best implement discussions regarding the consequences of diagnostic labelling required to minimise potential barriers.

### Implications

These results have implications for clinical encounters and health systems, and future research exploring these. Consumers had difficulty identifying non-cancer health conditions that could be identified through screening and did not recall GPs having conversations before screening regarding possible impacts of diagnostic labelling or screening procedures. By contrast, many GPs noted having discussions regarding screening procedures and test limitations before screening. This may simply reflect a lack of recall, or it might result from differences between GP and patient perceptions of needs and preferences regarding discussions related to screening. This finding, when combined with the literature on overestimating benefits and underestimating harms, indicates that this may be a problem.^25,26^ To address it, we may need to observe clinical encounters to determine whether and how these conversations occur.

A frequently occurring barrier to discussions, reported by both GPs and consumers, was time limitations. Careful balancing of time limitations with developed guidelines is important to ensure evidence-based healthcare. To facilitate this, changes to health systems, particularly in primary care, may be required to provide GPs sufficient time to engage in discussions with patients regarding the consequences of diagnostic labelling. In addition, consideration of the involvement of all health professionals who conduct screening tests in initiating conversations regarding diagnostic labelling may facilitate discussions while supporting time limitations. Although service provision time and cost require balancing, health system change may transform socially constructed views and understandings of health and healthcare.^14^ This transformation may facilitate how health conditions, diagnostic labels and intervention are viewed.

### Future research

We focused on non-cancer screening for individuals aged 40–65 years to align with guidelines for preventive health checks in Australia.^16^ However, research examining similarities and differences between older and younger age groups regarding screening is important, as health condition risk, treatment and prognosis may differ. GPs in our study expressed that patients had an implied understanding of non-cancer screening. Whether implied understanding is sufficient, or whether there is need for greater informed consent within non-cancer screening, remains unclear. It may be that discussion about the potential harms and benefits of a diagnostic label enhances patient informed consent. However additional research is required. It is possible that developing decision aids for screening tests, as well as patient-reported outcome measures and patient-reported experience measures, aimed at non-cancer screening and diagnostic labelling will improve this, but research is needed.^28,29^ Such further research would support the development of clinical guidelines to facilitate GP–patient interactions and minimise potential harms, while maximising potential benefits, when diagnostic labelling is required.

## Supporting information

Sims et al. supplementary material 1 Sims et al. supplementary material

Sims et al. supplementary material 2 Sims et al. supplementary material

## Data Availability

Data generated and/or analysed during the current study are available from the corresponding author upon reasonable request.

## Supplementary material

The supplementary material is available online at https://doi.org/10.1192/bjo.2025.5
