# Supplementary material for: Discussing the potential consequences of a diagnostic label before routine non-cancer screening: qualitative study with general practitioners and consumers
Source: BJPsych Open. 2025 Jun 11;11(4):e106. doi: 10.1192/bjo.2025.5 (PMC12188229; doi:10.1192/bjo.2025.5)
Supplement: Sims et al. supplementary material 1 — Sims et al. supplementary material [file S2056472425000055sup001.docx]

**Supplementary Table 1.** Questions posed in Semi-Structured Interviews with General Practitioners and Focus Groups with Consumers.

| **Semi-Structured Interviews** | **Focus Groups** |
| --- | --- |
| **Introduction**  Rebecca is Clinical Psychologists and PhD candidate at the Institute for Evidence-Based Healthcare at Bond University.  This research came about because we knew that labels have an influence, however, we weren’t sure of the when, how, in what contexts, and for whom labels really mattered. So, we’re trying to explore this more deeply through this study.  **Screening**   1. Regarding routine screening for non-cancer health conditions, what do you believe to be the:    1. Benefits?    2. Harms? 2. Can you please describe how you would communicate the potential consequences associated with routine screening of non-cancer conditions to a patient.   **Diagnostic Labelling**   1. What are your perceptions of the benefits/harms of providing diagnostic labels:    1. Generally?    2. Following routine screening or for low risk/mild conditions? 2. How do you communicate mild/ low risk health conditions (and diagnoses) to patients? 3. Do you have a case example of this? 4. How do you perceive such benefits/harms of such diagnoses to be received and understood by patients?   **Presentation**  Presentation of findings from a qualitative systematic scoping review^10^ and a quantitative systematic review^11^ regarding the consequences of diagnostic labelling. Recorded by RS and available at <https://osf.io/yp5wz>).  **Ending questions**   1. Did you have any questions or comments about the presentation? 2. Given the information provided in the presentations:    1. Regarding providing diagnostic labels for asymptomatic/low risk/ mild conditions, what are your perceptions of the:       1. Benefits?       2. Harms? 3. In the case example/s you provided, would you do anything differently? 4. How relevant is the information in the presentations to your patients prior to routine screening and/or diagnosis of low risk/ mild health conditions? Would you discuss this information with your patient prior to screening and if so, how would you potentially discuss this information? 5. Do you believe that discussion about the possible psychological benefits/harms of diagnostic labelling could assist in minimising negative, and maximising positive, impacts of diagnostic labelling following routine screening and/ or for low risk/ mild health conditions? 6. From the information discussed today, do you think you will make any changes to your clinical practice related to    1. Routine non-cancer screening    2. Communicating diagnostic labels for low risk/ mild health conditions 7. If you anticipate changes to your clinical practice, what might these be?   **Closing**  Do you have any closing comments, thoughts, or questions? | **Introduction**  Rebecca is Clinical Psychologists and PhD candidate at the Institute for Evidence-Based Healthcare at Bond University. Also facilitating today is Associate Professor Dr Rae Thomas, a psychologist and researcher.  This research came about because we knew that labels have an influence, however, we weren’t sure of the when, how, in what contexts, and for whom labels really mattered. So, we’re trying to explore this more deeply through this study.  **Presentations**   1. Presentation defining routine non-cancer screening and low risk/mild health conditions. Recorded by PG and available at <https://osf.io/75mpa>. 2. Presentation of findings from a qualitative systematic scoping review^10^ and a quantitative systematic review^11^ regarding the consequences of diagnostic labelling. Recorded by RS and available at <https://osf.io/yp5wz>.   *Opportunity for discussion after each presentation, with discussion prompts including:*   - *Have you thought about this information before?* - *Is this new information?* - *Where might this information apply in your healthcare?*   **Discussion**   1. If you have undergone routine screening for non-cancer health conditions, did your GP discuss the possible impacts of diagnostic labelling with you?    1. If yes, how did these discussions occur, and did you perceive them as beneficial to your decision making and psychological wellbeing?    2. If no, do you believe such discussions would have been beneficial to your decision making and psychological wellbeing? 2. If you have not undergone routine screening for non-cancer health conditions, was this because such tests have not been offered, or due to discussion with your GP which influenced your decision making? 3. Given the information provided in the presentations:    1. What is your understanding of the purpose of routine screening for non-cancer health conditions?    2. Regarding routine health condition screening, what are your perceptions of the:       1. Benefits?       2. Harms?    3. Regarding providing diagnostic labels for asymptomatic/low risk/ mild condition, what are your perceptions of the:       1. Benefits?       2. Harms? 4. Do you think the information provided in the presentations is relevant and should be discussed in the clinical encounter between the patient and GP prior to routine screening? Should this be discussed prior to diagnosis of low risk/ mild health conditions? 5. Do you believe that discussion about the possible psychological benefits/harms of diagnostic labelling could assist in minimising negative, and maximising positive, impacts of diagnostic labelling following routine screening and/ or for low risk/ mild health conditions? 6. Do you think the information discussed today will impact how you communicate with your healthcare providers?    1. If so, how?   **Closing**  Do you have any closing comments, thoughts, or questions? |
