# Supplementary material for: Discussing the potential consequences of a diagnostic label before routine non-cancer screening: qualitative study with general practitioners and consumers
Source: BJPsych Open. 2025 Jun 11;11(4):e106. doi: 10.1192/bjo.2025.5 (PMC12188229; doi:10.1192/bjo.2025.5)
Supplement: Sims et al. supplementary material 2 — Sims et al. supplementary material [file S2056472425000055sup002.docx]

**Supplementary Table 2.** Consolidated Criteria for Reporting Qualitative Research (COREQ).

| **Element** | **Item** | **Description** | **Where Reported** |
| --- | --- | --- | --- |
| **Domain 1: Research Team and Reflexivity** | | | |
| **Personal Characteristics** | | | |
| Interviewer/facilitator | 1 | Which author/s conducted the interview or focus group? | Procedures and Materials |
| Credentials | 2 | What were the researcher’s credentials? e.g., PhD, MD | The Research Team |
| Occupation | 3 | What was their occupation at the time of the study? | The Research Team |
| Gender | 4 | Was the researcher male or female? | The Research Team |
| Experience and training | 5 | What experience or training did the researcher have? Relationship with participants | The Research Team |
| Relationship established | 6 | Was a relationship established prior to study commencement? | Participants and Recruitment |
| Participant knowledge of the interviewer | 7 | What did the participants know about the researcher? e.g., personal goals, reasons for doing the research | Supp Table 1 |
| Interviewer characteristics | 8 | What characteristics were reported about the interviewer/facilitator? e.g., Bias, assumptions, reasons, and interests in the research topic | The Research Team |
| **Domain 2: Study Design** | | | |
| **Theoretical Framework** | | | |
| Methodological Orientation and Theory | 9 | What methodological orientation was stated to underpin the study? e.g., grounded theory, discourse analysis, ethnography, phenomenology, content analysis | Analyses |
| **Participant Selection** | | | |
| Sampling | 10 | How were participants selected? e.g., purposive, convenience, consecutive, snowball | Participants and Recruitment |
| Method of approach | 11 | How were participants approached? e.g., face-to-face, telephone, mail, email | Results |
| Sample size | 12 | How many participants were in the study? | Results |
| Non-participation | 13 | How many people refused to participate or dropped out? Reasons? | Results |

| **Setting** | | | |
| --- | --- | --- | --- |
| Setting of data collection | 14 | Where was the data collected? e.g., home, clinic, workplace | Procedure and Materials |
| Presence of non-participants | 15 | Was anyone else present besides the participants and researchers? | Procedure and Materials |
| Description of sample | 16 | What are the important characteristics of the sample? e.g., demographic data, date | Results |
| **Data Collection** | | | |
| Interview guide | 17 | Were questions, prompts, guides provided by the authors? Was it pilot tested? | Supp Table 1 |
| Repeat interviews | 18 | Were repeat interviews carried out? If yes, how many? | N/A |
| Audio/visual recording | 19 | Did the research use audio or visual recording to collect the data? | Procedure and Materials |
| Field notes | 20 | Were field notes made during and/or after the interview or focus group? | No |
| Duration | 21 | What was the duration of the interviews or focus group? | Procedure and Materials |
| Data saturation | 22 | Was data saturation discussed? | Analyses |
| Transcripts returned | 23 | Were transcripts returned to participants for comment and/or correction? | No |
| **Domain 3: analysis and findings** | | | |
| **Data Analysis** | | | |
| Number of data coders | 24 | How many data coders coded the data? | Analyses |
| Description of the coding tree | 25 | Did authors provide a description of the coding tree? | Figure 1 |
| Derivation of themes | 26 | Were themes identified in advance or derived from the data? | Analyses |
| Software | 27 | What software, if applicable, was used to manage the data? | Analyses |
| Participant checking | 28 | Did participants provide feedback on the findings? | No |
| **Reporting** | | | |
| Quotations presented | 29 | Were participant quotations presented to illustrate the themes / findings? Was each quotation identified? e.g., participant number | Table 4 and Table 5 |
| Data and findings consistent | 30 | Was there consistency between the data presented and the findings? | Results, Figure 1, Tables 3-5 |
| Clarity of major themes | 31 | Were major themes clearly presented in the findings? | Figure 1 and Table 3 |
| Clarity of minor themes | 32 | Is there a description of diverse cases or discussion of minor themes | Results |
